# Summary of the National Advisory Committee on Immunization (NACI) Seasonal Influenza Vaccine Statement for 2024–2025

**DOI:** 10.14745/ccdr.v50i11a01

**Published:** 2024-11-07

**Authors:** Anabel Gil, Winnie Siu, Jesse Papenburg

**Affiliations:** 1 Centre for Immunization Surveillance and Programs, Public Health Agency of Canada, Ottawa, ON; 2 School of Epidemiology and Public Health, Faculty of Medicine, University of Ottawa, ON; 3 NACI Influenza Working Group Chair; 4 Division of Pediatric Infectious Diseases, Department of Pediatrics, Montréal Children’s Hospital of the McGill University Health Centre, Montréal, QC; 5 Division of Microbiology, Department of Clinical Laboratory Medicine, OPTILAB Montréal - McGill University Health Centre, Montréal, QC; 6 Department of Epidemiology, Biostatistics and Occupational Health, School of Population and Global Health, McGill University, Montréal, QC

**Keywords:** National Advisory Committee on Immunization, NACI, influenza, influenza vaccine, guidance

## Abstract

**Background:**

The National Advisory Committee on Immunization (NACI) reviews the evolving evidence on influenza immunization and provides annual recommendations regarding the use of seasonal influenza vaccines. The *NACI Statement on Seasonal Influenza Vaccine for 2024–2025* updates the NACI recommendations from the previous year.

**Objective:**

To summarize the 2024–2025 NACI seasonal influenza vaccine recommendations and to highlight new and updated information.

**Methods:**

For the development of the *Statement on Seasonal Influenza Vaccine for 2024–2025*, the NACI Influenza Working Group applied the NACI evidence-based process to assess available evidence and formulate recommendations. These recommendations underwent a thorough evaluation and were approved by NACI based on the available evidence.

**Results:**

Key updates for the 2024–2025 influenza season include updated immunization recommendations reflecting changes in influenza epidemiology and revised guidance for vaccine administration during pregnancy and in older adults.

**Conclusion:**

The National Advisory Committee on Immunization recommends that any age-appropriate quadrivalent or trivalent influenza vaccine should be used for individuals six months of age and older who do not have contraindications or precautions. NACI reaffirms the importance of influenza vaccination with inactivated or recombinant influenza vaccines in pregnancy. Finally, NACI recommends that inactivated high-dose (IIV-HD), inactivated adjuvanted (IIV-Adj) or recombinant influenza vaccine (RIV) should be offered, when available, over other influenza vaccines for adults 65 years of age and older.

## Introduction

Each year, Canada experiences seasonal influenza epidemics, primarily during late fall and winter. The severity of these outbreaks fluctuates annually due to factors such as circulating virus types and affected populations ((1)). On average, Canada sees approximately 12,200 influenza-related hospitalizations and 3,500 influenza-related deaths annually ((2,3)). Vaccination remains the most effective defence against influenza and its complications.

The National Advisory Committee on Immunization (NACI) provides the Public Health Agency of Canada (PHAC) with annual recommendations on the use of authorized seasonal influenza vaccines, reflecting shifts in epidemiology, immunization practices and available products in Canada. The NACI Influenza Working Group leads the annual update of the NACI Statement on Seasonal Influenza Vaccine, involving a thorough review and evaluation of the literature as well as discussion and debate at the scientific and clinical practice levels. On July 25, 2024, PHAC released new guidance from NACI on the use of seasonal influenza vaccines for the 2024–2025 season, which is based on current evidence and expert opinion. This article provides a concise summary of NACI’s recommendations and supporting information for the 2024–2025 influenza season, with emphasis on new or updated information since the *Statement on Seasonal Influenza Vaccine for 2023–2024*. Conclusions from evidence reviews on the use of influenza vaccine during pregnancy and in older adults are also presented. Additionally, the article addresses ongoing research following recent outbreaks of highly pathogenic avian influenza (HPAI) A(H5N1). For detailed information, refer to the new *NACI Advisory Committee Statement on Seasonal Influenza Vaccine for 2024–2025* (the Statement) on the PHAC website ((4)).

## Methods

In the preparation of the 2024–2025 seasonal influenza vaccine recommendations, NACI’s Influenza Working Group identified the need for evidence reviews for new topics, analyzed available evidence and developed updated recommendations using NACI’s evidence-based process. Further details regarding the strength of NACI recommendations are available in **Table A1** in the **Appendix**. NACI’s peer-reviewed framework and evidence-informed tools (including the Ethics Integrated Filters, Equity Matrix, Feasibility Matrix and Acceptability Matrix) were applied to help ensure that issues related to ethics, equity, feasibility and acceptability are systematically assessed and integrated into NACI guidance ((5)).

The 2024–2025 Statement includes an addendum ((6)) with updated guidance on influenza immunization in response to the global absence of B/Yamagata viruses. Recommendations and supporting evidence from 1) *Updated Guidance on Influenza Vaccination During Pregnancy* ((7)) and 2) *Supplemental Guidance on Influenza Vaccination in Adults 65 Years of Age and Older* ((8)), both published since the 2023–2024 Statement, were also integrated.

For more information on NACI recommendations related to seasonal influenza vaccination, please see the Canadian Immunization Guide chapter on influenza vaccines ((9)), as well as additional statements on the NACI web page.

## Results

### New or updated information for 2024–2025

**Addendum to the 2024–2025 Statement:** The 2024–2025 Statement includes an addendum with updated information and guidance on influenza vaccines, reflecting the global absence of B/Yamagata viruses since March 2020. NACI supports transitioning from quadrivalent to trivalent influenza vaccines based on this change in epidemiology, the theoretical risk of reintroduction of B/Yamagata viruses with the continued production and use of quadrivalent vaccines, and World Health Organization (WHO) recommendations and expert consensus. Therefore, NACI has removed its preferential recommendation for quadrivalent vaccines in children, and now, **NACI recommends that any age-appropriate quadrivalent or trivalent influenza vaccine should be used for individuals six months of age and older who do not have contraindications or precautions.** For the 2024–2025 influenza season in Canada, vaccine availability is anticipated to remain unchanged, with quadrivalent formulations continuing to be supplied for public programs. Adjuvanted inactivated influenza vaccines will remain trivalent and continue to be available in Canada. Please refer to the *Addendum to the NACI Statement on Seasonal Influenza Vaccine for 2024–2025 – Transition from Quadrivalent to Trivalent Influenza Vaccines* for more information ((6)).

For the 2023–2024 influenza season, NACI reviewed the available evidence and developed updated recommendations for the following topics:

• The use of influenza vaccines during pregnancy: NACI continues to **strongly recommend that inactivated or recombinant influenza vaccines be offered during pregnancy, at any gestational age.** NACI also continues to include pregnant individuals among those for whom influenza vaccination is particularly important. Finally, NACI reaffirms its recommendation that influenza vaccination may be given at the same time as, or at any time before or after administration of another vaccine, including the COVID-19 or pertussis vaccine.

For complete details of this review, rationale, relevant considerations and additional information supporting this recommendation, refer to the *Updated Guidance on Influenza Vaccination During Pregnancy* ((7)).

• The use of influenza vaccines in older adults: NACI strongly recommends that **inactivated high-dose (IIV-HD), inactivated adjuvanted (IIV-Adj) or recombinant influenza vaccine (RIV) should be offered, when available, over other influenza vaccines for adults 65 years of age and older.** If a preferred product is not available, any of the available age-appropriate influenza vaccines should be used.

For complete details of this review, rationale, relevant considerations and additional information supporting this recommendation, refer to the *Supplemental Guidance on Influenza Vaccination in Adults 65 Years of Age and Older* ((8)).

**Seasonal influenza guidance in the context of highly pathogenic avian influenza (HPAI) A(H5N1) outbreaks:** Due to recent HPAI A(H5N1) outbreaks in poultry and mammals, including, as of July 2024, multiple cases in the United States of bird-to-human and cow-to-human transmissions, NACI reiterates its recommendation that all individuals six months of age and older should receive a seasonal influenza vaccine. This includes those likely to have significant risk of exposure to influenza A(H5N1) through interactions with birds or mammals (such as poultry, livestock, slaughterhouse and processing plant workers, wildlife officers/researchers and veterinarians). NACI will be conducting an evidence review to determine if there is a need to permanently expand its list of individuals for whom influenza vaccination is particularly important, beyond the current group, to others at high risk of exposure to circulating A(H5N1) viruses.

More information can be found in the *Important notice 2: Outbreaks of highly pathogenic avian influenza in Canada and U.S.* section of the *Seasonal Influenza Vaccine for 2024–2025* on the PHAC website ((4)).

### Summary of National Advisory Committee on Immunization recommendations for the use of influenza vaccines for the 2024–2025 influenza season

The National Advisory Committee on Immunization recommends that any age-appropriate quadrivalent or trivalent influenza vaccine should be used for individuals six months of age and older who do not have contraindications or precautions. Vaccination should be offered as a priority to people at high risk of influenza-related complications or hospitalization, people capable of transmitting influenza to those at high risk of complications and others as indicated in **List 1**:

• Both quadrivalent and trivalent formulations are clinically safe and effective

• Since March 2020, B/Yamagata viruses have not been detected globally, leading to the recommendation to exclude the B/Yamagata component from 2024 to 2025 influenza vaccines, aligning with WHO guidance

• Previously, quadrivalent vaccines were preferred for children due to the presence of both influenza B components, and NACI now has no preference between quadrivalent and trivalent vaccines

**List 1: Groups for whom influenza vaccination is particularly important^a^**

**Table:** 

**People at high risk of influenza-related complications or hospitalization:** **• All children 6–59 months of age** **• Adults and children with the following chronic health conditions^b^:** **o Cardiac or pulmonary disorders (including bronchopulmonary dysplasia, cystic fibrosis and asthma)** **o Diabetes mellitus and other metabolic diseases** **o Cancer, immune compromising conditions (due to underlying disease, therapy, or both, such as solid organ transplant or hematopoietic stem cell transplant recipients)** **o Renal disease** **o Anemia or hemoglobinopathy** **o Neurologic or neurodevelopmental conditions (includes neuromuscular, neurovascular, neurodegenerative, neurodevelopmental conditions and seizure disorders [and, for children, includes febrile seizures and isolated developmental delay], but excludes migraines and psychiatric conditions without neurological conditions)^c^** **o Morbid obesity (defined as BMI of 40 kg/m^2^ and over)** **o Children six months to 18 years of age undergoing treatment for long periods with acetylsalicylic acid, because of the potential increase of Reye syndrome associated with influenza** **• All individuals who are pregnant** **• All individuals of any age who are residents of nursing homes and other chronic care facilities** **• Adults 65 years of age and older** **• Indigenous Peoples** **People capable of transmitting influenza to those at high risk:** **• Healthcare and other care providers in facilities and community settings who, through their activities, are capable of transmitting influenza to those at high risk** **• Household contacts, both adults and children, of individuals at high risk, whether or not the individual at high risk has been vaccinated:** **o Household contacts of individuals at high risk** **o Household contacts of infants younger than six months of age, as these infants are at high risk but cannot receive influenza vaccine** **o Members of a household expecting a newborn during the influenza season** **• Those providing regular childcare to children newborn to 59 months of age, whether in or out of the home** **• Those who provide services within closed or relatively closed settings to people at high risk (e.g., crew on a cruise ship)** **Others:** **• People who provide essential community services** **• People who are in direct contact with poultry infected with avian influenza during culling operations**

^a^ List reproduced from *NACI Seasonal Influenza Vaccine Statement for 2024–2025* ((4))^b^ Refer to *Immunization of Persons with Chronic Diseases* ((10)) and *Immunization of Immunocompromised Persons* ((11)) in Part 3 of the Canadian Immunization Guide for additional information about vaccination of people with chronic diseases^c^ Refer to the *NACI Statement on Seasonal Influenza Vaccine for 2018–2019* ((12)) for rationale supporting the decision to include persons with neurologic or neurodevelopment conditions among the groups for whom influenza vaccination is particularly important and the *Literature Review on Individuals with Neurologic or Neurodevelopment Conditions and Risk of Serious Influenza-Related Complications* (13)) for additional details of the evidence reviews that were conducted

### Recommendations on choice of influenza vaccine type for individual and public health program-level decision making by age group

• Children aged six to 23 months can receive IIV-Adj, standard-dose inactivated influenza vaccine (IIV-SD) and mammalian cell culture based inactivated influenza vaccine (IIV-cc).

o Influvac Tetra (IIV4-SD) is not recommended for those younger than three years due to insufficient evidence.

• Children six to 17 years can receive IIV-SD, IIV-cc and live attenuated influenza vaccine (LAIV).

o LAIV is suitable for children with stable, non-severe asthma, cystic fibrosis (without immunosuppressive drug treatment) and stable HIV infection (if being treated with antiretroviral therapy and has adequate immune function).

o LAIV should not be used in children or adolescents with contraindications or precautions, including severe asthma, medically attended wheezing within the past seven days, current receipt of aspirin or aspirin-containing therapy and immune compromising conditions. Stable HIV infection is an exception, provided the child has been on highly active antiretroviral therapy for at least four months with adequate immune function. Live attenuated influenza vaccine should also not be used in pregnancy; IIV-SD or IIV-cc are preferred choices during pregnancy.

o IIV4-SD is not recommended for those younger than three years due to insufficient evidence.

• Adults 18 to 59 years can receive IIV-SD, IIV-cc, RIV and LAIV; however, inactivated influenza vaccine may provide better protection than LAIV in healthy adults.

o LAIV is not recommended for adults with any chronic health conditions listed in List 1 (including immune compromising conditions) and healthcare workers. LAIV is also not recommended in pregnancy; IIV-SD, IIV-cc or RIV are preferred choices during pregnancy.

• Adults 60 to 64 years can receive IIV-SD, IIV-cc and RIV.

• Adults 65 years of age and older should preferentially receive IIV-HD, IIV-Adj or RIV, when available, over IIV-SD and IIV-cc. If a preferred product is not available, any age-appropriate influenza vaccine should be used.

For more information, refer to the *Addendum to the NACI Statement on Seasonal Influenza Vaccine for 2024–2025 – Transition from Quadrivalent to Trivalent Influenza Vaccines* ((6)). The Canadian Immunization Guide Chapter on Influenza Vaccines has also been updated accordingly.

Recommended dose and route of administration of influenza vaccine types by age, are summarized in **Table 1**.

**Table 1:** Recommended dose and route of administration, by age, for influenza vaccine types authorized for the 2024–2025 influenza season^a^

Age group	Influenza vaccine type (route of administration)						Number of doses required
	IIV-SD^b^ (IM)	IIV-cc^c^ (IM)	IIV-Adj^d^ (IM)	IIV-HD^e^ (IM)	RIV^f^ (IM)	LAIV^g^ (intranasal)	
6–23 months^h^	0.5 mL^i^	0.5 mL	0.25 mL	-	-	-	1 or 2^j^
2–8 years	0.5 mL	0.5 mL	-	-	-	0.2 mL (0.1 mL per nostril)	1 or 2^j^
9–17 years	0.5 mL	0.5 mL	-	-	-	0.2 mL (0.1 mL per nostril)	1
18–59 years	0.5 mL	0.5 mL	-	-	0.5 mL	0.2 mL (0.1 mL per nostril)	1
60–64 years	0.5 mL	0.5 mL	-	-	0.5 mL	-	1
65 years and older	0.5 mL	0.5 mL	0.5 mL	0.7 mL	0.5 mL	-	1

Abbreviations: IIV-Adj, adjuvanted inactivated influenza vaccine; IIV-cc, mammalian cell culture based inactivated influenza vaccine; IIV-HD, high-dose inactivated influenza vaccine; IIV-SD, standard-dose inactivated influenza vaccine; IM, intramuscular; LAIV, live attenuated influenza vaccine; RIV, recombinant influenza vaccine

^a^ Table reproduced from *National Advisory Committee on Immunization Seasonal Influenza Vaccine Statement for 2024–2025* ((4))

^b^ Afluria® Tetra (five years and older), Flulaval® Tetra (six months and older), Fluzone® Quadrivalent (six months and older), Influvac® Tetra (three years and older)

^c^ Flucelvax® Quad (six months and older)

^d^ Fluad Pediatric® (6–23 months) or Fluad® (65 years and older)

^e^ Fluzone® High-Dose Quadrivalent (65 years and older)

^f^ Supemtek™ (18 years and older)

^g^ FluMist® Quadrivalent (2–59 years)

^h^ There is insufficient evidence for recommending vaccination with Influvac Tetra (IIV4-SD) in children younger than three years of age

^i^ Evidence suggests moderate improvement in antibody response in infants, without an increase in reactogenicity, with the use of full-vaccine doses (0.5 mL) for unadjuvanted inactivated influenza vaccines. This moderate improvement in antibody response without an increase in reactogenicity is the basis for the full dose recommendation for unadjuvanted inactivated vaccine for all ages. For more information, refer to *Statement on Seasonal Influenza Vaccine for 2011–2012* ((14))

^j^ Children six months to less than nine years of age receiving seasonal influenza vaccine for the first time in their life should be given two doses of influenza vaccine, with a minimum interval of four weeks between doses. Children six months to less than nine years of age who have been properly vaccinated with one or more doses of seasonal influenza vaccine in the past should receive one dose of influenza vaccine per season thereafter

## Conclusion

The National Advisory Committee on Immunization continues to recommend annual influenza vaccination for all individuals aged six months and older (noting product-specific age indications and contraindications). Influenza vaccination is particularly important for people at high risk of influenza-related complications or hospitalization, people capable of transmitting influenza to those at high risk, people who provide essential community services and people in direct contact during culling operations with poultry infected with avian influenza. For the 2024–2025 influenza season, NACI: 1) recommends that any age-appropriate quadrivalent or trivalent influenza vaccine should be used for individuals six months of age and older who do not have contraindications or precautions; 2) continues to strongly recommend that inactivated or recombinant influenza vaccines be offered during pregnancy, at any gestational age; and 3) strongly recommends that IIV-HD, IIV-Adj or RIV should be offered, when available, over other influenza vaccines for adults 65 years of age and older.

## Appendix

**Table A1:** Strength of the National Advisory Committee on Immunization recommendations

Strength of NACI recommendation (based on factors not isolated to strength of evidence, e.g., public health need)	Strong	Discretionary
Wording	“should/should not be offered”	“may be considered“
Rationale	Known/anticipated advantages outweigh known/anticipated disadvantages (“should“) OR known/anticipated disadvantages outweigh known/anticipated advantages (“should not“)	Known/anticipated advantages closely balanced with known/anticipated disadvantages OR uncertainty in the evidence of advantages and disadvantages exists
Implication	A strong recommendation applies to most populations/individuals and should be followed unless a clear and compelling rationale for an alternative approach is present	A discretionary recommendation may be considered for some populations/individuals in some circumstances Alternative approaches may be reasonable

Abbreviation: NACI, National Advisory Committee on Immunization

## References

[1] World Health Organization. Estimating disease burden of influenza. Geneva, CH: WHO; 2021. https://www.who.int/europe/activities/estimating-disease-burden-of-influenza

[2] Schanzer DL, McGeer A, Morris K. Statistical estimates of respiratory admissions attributable to seasonal and pandemic influenza for Canada. Influenza Other Respir Viruses 2013;7(5):799–808. 10.1111/irv.12011 23122189 PMC3796862

[3] Schanzer DL, Sevenhuysen C, Winchester B, Mersereau T. Estimating influenza deaths in Canada, 1992-2009. PLoS One 2013;8(11):e80481. 10.1371/journal.pone.0080481 24312225 PMC3842334

[4] National Advisory Committee on Immunization. Statement on seasonal influenza vaccine for 2024–2025. Ottawa, ON: PHAC; 2024. https://www.canada.ca/en/public-health/services/publications/vaccines-immunization/national-advisory-committee-immunization-statement-seasonal-influenza-vaccine-2024-2025.html 10.14745/ccdr.v50i11a01 PMC11542546 39525078

[5] Ismail SJ, Hardy K, Tunis MC, Young K, Sicard N, Quach C. A framework for the systematic consideration of ethics, equity, feasibility, and acceptability in vaccine program recommendations. Vaccine 2020;38(36):5861–76. 10.1016/j.vaccine.2020.05.051 32532544 PMC7283073

[6] National Advisory Committee on Immunization. Addendum to the statement on seasonal influenza vaccine for 2024-2025: Transition from quadrivalent to trivalent influenza vaccines. Ottawa, ON: PHAC; 2024. https://www.canada.ca/en/public-health/services/publications/vaccines-immunization/national-advisory-committee-immunization-statement-addendum-seasonal-influenza-vaccine-2024-2025.html

[7] National Advisory Committee on Immunization. Updated guidance on influenza vaccination during pregnancy. Ottawa, ON: PHAC; 2023. https://www.canada.ca/en/public-health/services/publications/vaccines-immunization/national-advisory-committee-immunization-updated-guidance-influenza-vaccination-during-pregnancy.html 10.14745/ccdr.v50i34a01 PMC11073834 38716409

[8] National Advisory Committee on Immunization. Supplemental guidance on influenza vaccination in adults 65 years of age and older. Ottawa, ON: PHAC; 2024. https://www.canada.ca/en/public-health/services/publications/vaccines-immunization/national-advisory-committee-immunization-supplemental-guidance-influenza-vaccination-adults-65-years-older.html 10.14745/ccdr.v50i11a02 PMC11542547 39525077

[9] Public Health Agency of Canada. Influenza vaccines: Canadian Immunization Guide. Ottawa, ON: PHAC; 2024. https://www.canada.ca/en/public-health/services/publications/healthy-living/canadian-immunization-guide-part-4-active-vaccines/page-10-influenza-vaccine.html

[10] Public Health Agency of Canada. Immunization of persons with chronic diseases: Canadian Immunization Guide. Ottawa, ON: PHAC; 2024. https://www.canada.ca/en/public-health/services/publications/healthy-living/canadian-immunization-guide-part-3-vaccination-specific-populations/page-7-immunization-persons-with-chronic-diseases.html

[11] Public Health Agency of Canada. Immunization of immunocompromised persons: Canadian Immunization Guide. Ottawa, ON: PHAC; 2024. https://www.canada.ca/en/public-health/services/publications/healthy-living/canadian-immunization-guide-part-3-vaccination-specific-populations/page-8-immunization-immunocompromised-persons.html

[12] National Advisory Committee on Immunization. Canadian Immunization Guide Chapter on Influenza and Statement on Seasonal Influenza Vaccine for 2018–2019. Ottawa, ON: PHAC; 2018. https://www.canada.ca/en/public-health/services/publications/healthy-living/canadian-immunization-guide-statement-seasonal-influenza-vaccine-2018-2019.html

[13] National Advisory Committee on Immunization. Literature Review on Individuals with Neurologic or Neurodevelopment Conditions and Risk of Serious Influenza-Related Complications - Executive Summary. Ottawa, ON: PHAC; 2018. https://www.canada.ca/en/public-health/services/publications/healthy-living/executive-summary-literature-review-individuals-neurologic-neurodevelopment-conditions-risk-serious-influenza-related-complications.html

[14] National Advisory Committee on Immunization. NACI Statement: Seasonal Influenza Vaccine, 2011-2012. Ottawa, ON: PHAC; 2011. https://www.canada.ca/en/public-health/services/reports-publications/canada-communicable-disease-report-ccdr/monthly-issue/2011-37/canada-communicable-disease-report-acs-5.html 10.14745/ccdr.v37i00a05 PMC6802429 31682646

